# Prognostic Value of lncRNA PVT1 for Patients with Gastric Cancer: A Meta-Analysis

**DOI:** 10.1155/2021/5595965

**Published:** 2021-12-02

**Authors:** Jinyong Hao, Bo Yuan, Yani Gou, Jichun Ma, Xiaojun Huang

**Affiliations:** ^1^Department of Gastroenterology, Lanzhou University Second Hospital, Lanzhou, China; ^2^Gansu Provincial Digestive Endoscopy Engineering Research Center, Lanzhou, China; ^3^Department of General Surgery, Lanzhou University Second Hospital, Lanzhou, China; ^4^First Clinical Medical College, Lanzhou University, Lanzhou, China

## Abstract

**Objective:**

To evaluate the prognostic value of lncRNA PVT1 for patients with gastric cancer.

**Methods:**

A comprehensive literature searching was performed in PubMed, Cochrane Library, Web of Science, Embase, CNKI, CBM, and Wanfang Database to identify published studies on the expression level of lncRNA PVT1 in human gastric cancer. STATA 12.0 was conducted to perform the meta-analysis. Clinical outcomes including patients' age, genders, TNM stage, OS, and DFS were assessed in the study.

**Results:**

A total of 8 studies involving 747 patients were included in this meta-analysis. The results of meta-analysis showed that higher expression level of lncRNA PVT1 was associated with GC patients' gender (for male: OR = 2.27, 95% CI: 1.67~3.07, *P* = 0.000), invasion depth (for T3~4: OR = 3.98, 95% CI: 2.85~5.56, *P* = 0.000), poorer OS (HR = 1.68, 95% CI: 1.43~1.97, *P* = 0.000), and DFS (HR = 1.74, 95% CI: 1.44~2.08, *P* = 0.000).

**Conclusion:**

Higher expression level of lncRNA PVT1 is significantly associated with GC patients' gender, invasion depth, poorer OS, and worse DFS. lncRNA PVT1 might act as a novel predictive biomarker of poor prognosis and clinicopathological characteristics for gastric cancer.

## 1. Introduction

Long noncoding RNAs (lncRNAs) are a class of RNA molecules with a length of more than 200 nucleotides [1]. Due to the lack of open reading frames, they cannot encode proteins. In the early stage, they were considered as “junk sequences” in the process of gene transcription [2]. Although lncRNAs do not encode proteins, they can still regulate gene expression through a variety of mechanisms at the epigenetic, transcriptional, and posttranscriptional levels, which become one of the research hotspots in recent years [3]. A large number of studies have shown that lncRNAs play an important role in the invasion, migration, and metastasis of tumors [4, 5]. Among them, plasma tumor variant translocation 1 (PVT1) was reported as a key biomarker of prognosis for patients with gastric cancer (GC) [6].

Numerous studies have reported that PVT1 showed higher expression in gastric cancer tissues and cell lines, can promote the proliferation and invasion of GC cells, and is closely related to advanced tumor stage and lymph node metastasis [7, 8]. However, previous published studies have been limited by low study sample size; the association between clinical outcomes and PVT1 expression remains unclear. Thus, we conducted a meta-analysis with the aim to evaluate the prognostic value of PVT1 for patients with GC.

## 2. Methods

### 2.1. Search Strategy

A comprehensive literature searching was performed in PubMed, Cochrane Library, Web of Science, Embase, CNKI, CBM, and Wanfang Database to identify published studies on the expression level of lncRNA PVT1 in human gastric cancer in English and Chinese language. The following search terms were used in online databases: “gastric cancer”, “stomach cancer”, “gastric neoplasm”, “long non-coding RNA”, “lncRNA”, “plasma tumor variant translocation 1”, “PVT1”, “clinical outcome”, “prognosis”, “overall survival”, and “disease free survival”. The literature search was undertaken from databases' construction to April 30, 2020. The meta-analysis was conducted according to the guideline of the Preferred Reporting Items for Systematic Reviews and Meta-Analysis (PRISMA).

### 2.2. Inclusion Criteria

Two reviewers screened and selected all search records on publication titles, abstracts, and full manuscript. Inclusion criteria were as follows: (1) all studies were published in English and Chinese, and full text is available on the online databases; (2) patients were diagnosed with gastric cancer in any TNM stage; (3) the expression level of lncRNA PVT1 was reported in studies, and lncRNA PVT1 was detected by established molecular methods, such as qRT-PCR and western blot; (4) studies included in meta-analysis demonstrated the correlation between lncRNA PVT1 expression level and GC patients' clinical characteristics, survival data, and prognostic value; (5) sufficient statistical analysis was required, including overall survival (OS) and disease-free survival (DFS) and their hazard ratios (HRs) and 95% confidence interval (95% CI). Kaplan-Meier survival curves were also necessary.

### 2.3. Data Extraction

Two researchers extracted the required data from included studies, including (1) characteristics of included studies: study title, the year of publication, authors, country, tumor stage, sample size, cut-off value, follow-up duration, and lncRNA detection methods; (2) clinical outcomes: patients' age, genders, and TNM stage; and (3) survival data: OS, DFS, HR value, and 95% CIs.

### 2.4. Quality Assessment

The quality of included studies was evaluated according to the Newcastle-Ottawa Scale (NOS) [9] by two reviewers independently. Any disagreements were discussed and resolved by another reviewer. NOS score ranged from 0 to 9; it was regarded as a high quality if one is with a NOS score > 7.

### 2.5. Statistical Analysis

STATA 12.0 was conducted to perform the meta-analysis. Chi-based *Q*-test and *I*^2^ test were used to assess the statistical heterogeneity between studies. When *I*^2^ < 50% or *Q*-test *P* > 0.1, there is no heterogeneity in the data analysis; the fixed-effect model was adopted to perform the meta-analysis. Otherwise, the random-effect model was used in the meta-analysis. If the results are heterogeneous, subgroup analysis of possible factors that may lead to heterogeneity was undertaken. Sensitivity analysis was also conducted to assess the ability of the combined results and to determine the source of any heterogeneity. And Begg's test was also used to evaluate the publication bias.

## 3. Results

### 3.1. The Characteristics of Included Studies

**Figure 1:**
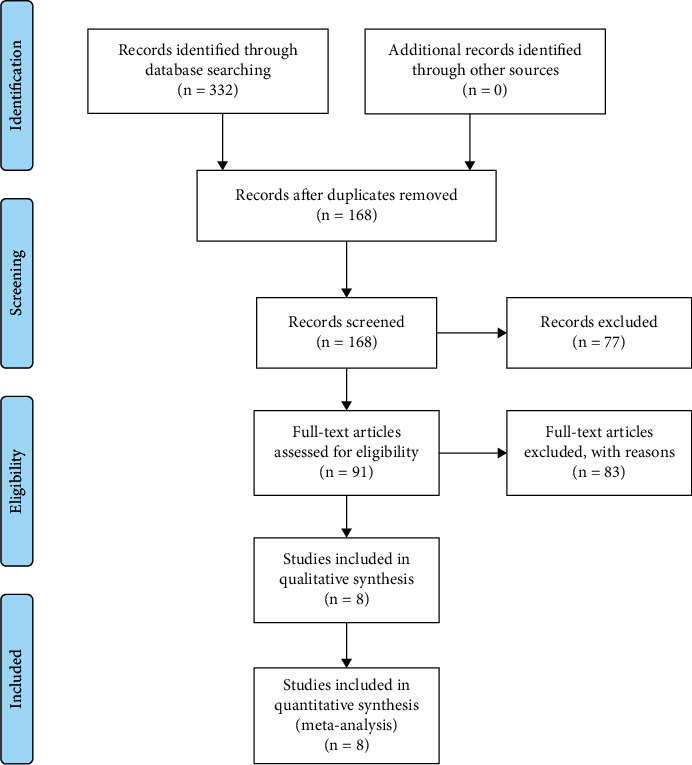
PRISMA flowchart of literature selection.

A systematic literature search was performed in databases. As shown in Figure 1, a total of 332 publications were identified from online databases. 164 duplicates were removed by Endnote X17, and 77 records were excluded after screening the titles and abstracts. 91 publications were dealt with reading the full text. Based on the inclusion criteria, 8 studies [10–17] were included in the present meta-analysis.

Of the eight studies on the association between expression level of lncRNA PVT1 and GC patients identified, 747 patients were included in this meta-analysis. All of the included studies are from China and reported the data on stage I-IV GC patients. Five of them reported the cut-off value, and the qRT-PCR method was used to detect the expression level of lncRNA PVT1 in all included publications. The results of quality assessment for included studies ranged from 7 to 8, which demonstrated a high quality for the 8 included publications (Table 1).

### 3.2. Association between lncRNA PVT1 Expression Level and GC Patients' Age

**Figure 2:**
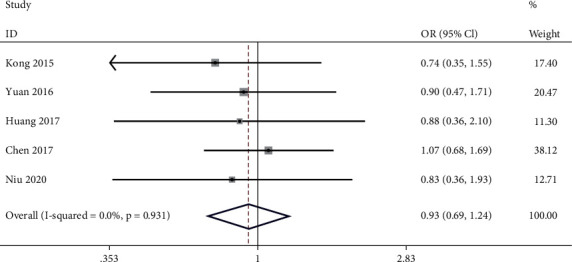
Meta-analysis of association between high expression level of lncRNA PVT1 and GC patients' age (>50 yrs vs. <50 yrs).

Five of the included studies reported the association between lncRNA PVT1 expression level and GC patients' age. No heterogeneity between studies was observed (*I*^2^ = 0.0%, *P* = 0.931). Meta-analysis in a fixed-effect model showed that there is no relationship between high expression level of lncRNA PVT1 and GC patients' age (OR = 0.93, 95% CI: 0.68~1.24, *P* = 0.606) (Figure 2).

### 3.3. Association between lncRNA PVT1 Expression Level and GC Genders

**Figure 3:**
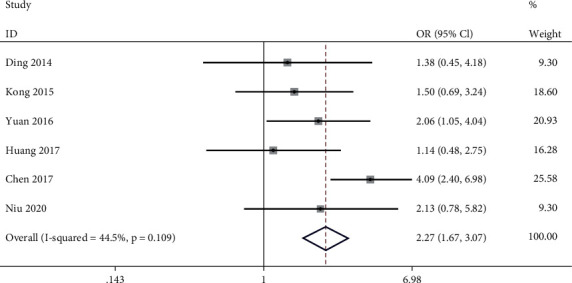
Meta-analysis of association between high expression level of lncRNA PVT1 and GC patients' genders (male vs. female).

Six studies reported the association between lncRNA PVT1 expression level and GC genders. There is no heterogeneity between studies (*I*^2^ = 44.5%, *P* = 0.109); a fixed-effect model was conducted to perform meta-analysis. The results showed that high expression level of lncRNA PVT1 was associated with male gender (OR = 2.27, 95% CI: 1.67~3.07, *P* = 0.000) (Figure 3).

### 3.4. Association between lncRNA PVT1 Expression Level and Invasion Depth (T3~4 vs. T1~2)

**Figure 4:**
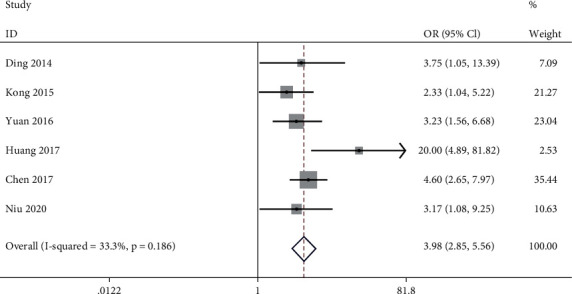
Meta-analysis of association between lncRNA PVT1 expression level and invasion depth (T3~4 vs. T1~2).

Six of the included studies reported association between lncRNA PVT1 expression level and invasion depth. Meta-analysis in a fixed-effect model (study heterogeneity: *I*^2^ = 33.3%, *P* = 0.186) showed that high expression level of lncRNA PVT1 was associated with T3~4 (OR = 3.98, 95% CI: 2.85~5.56, *P* = 0.000) (Figure 4).

### 3.5. Association between lncRNA PVT1 Expression Level and Overall Survival

**Figure 5:**
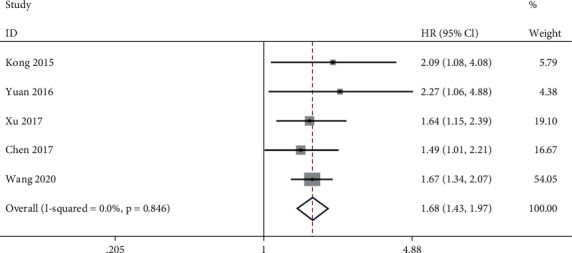
Meta-analysis of association between lncRNA PVT1 expression level and overall survival (high expression level vs. low expression level).

Five of the included studies reported association between lncRNA PVT1 expression level and overall survival. Meta-analysis in a fixed-effect model (*I*^2^ = 0.0%, *P* = 0.845) showed that higher expression level of lncRNA PVT1 is associated with poorer OS for patients with GC (HR = 1.68, 95% CI: 1.43~1.97, *P* = 0.000) (Figure 5).

### 3.6. Association between lncRNA PVT1 Expression Level and Disease-Free Survival

**Figure 6:**
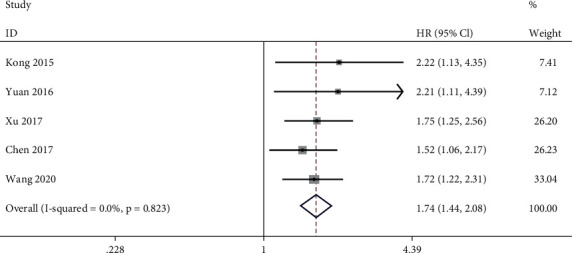
Meta-analysis of association between lncRNA PVT1 expression level and DFS (high expression level vs. low expression level).

Five of the included studies reported the association between lncRNA PVT1 expression level and DFS. Meta-analysis performed in a fixed-effect model (*I*^2^ = 0.0%, *P* = 0.823) showed that high expression level of lncRNA PVT1 was associated with poorer DFS for GC patients (HR = 1.74, 95% CI: 1.44~2.08, *P* = 0.000) (Figure 6).

## 4. Discussion

The length of lncRNA PVT1 is about 1716 NT, which is located in chromosome 8q24 region and sense strand [18]. It spans more than 300 KB genome interval [19]. In tumor cells, chromosome 8q24 region is the highest target of DNA copy number amplification, and its abnormal amplification often indicates high risk of cancer [20]. The abnormal expression of lncRNA PVT1 is associated with many human tumors, such as lung cancer, gastric cancer, colorectal cancer, breast cancer, pancreatic cancer, and prostate cancer [21–24]. Different expressions of lncRNA PVT1 in various tumors indicate that it plays an important role in the occurrence and development of tumors.

Gastric cancer is one of the most common gastrointestinal tumors, which is the third leading cause of cancer-related death and accounting for 9% of the global cancer mortality [25]. In 2015, the number of confirmed cases of gastric cancer in China was about 679000, only second to lung cancer (733000), higher than esophageal cancer (478000) and liver cancer (466000) [26]. Although great progress has been made in the surgical treatment and chemotherapy of gastric cancer in recent years, due to the lack of specific symptoms and signs in the early stage of gastric cancer, most patients with gastric cancer are in the advanced stage at the time of diagnosis, which seriously affects the prognosis of patients [27]. The 5-year survival rate of patients with advanced gastric cancer is less than 10% [28]. Therefore, it is urgent to find a new biomarker for the diagnosis and prognosis of gastric cancer.

In present studies, the results of meta-analysis showed that higher expression level of lncRNA PVT1 was associated with GC patients' gender (for male: OR = 2.27, 95% CI: 1.67~3.07, *P* = 0.000), invasion depth (for T3~4: OR = 3.98, 95% CI: 2.85~5.56, *P* = 0.000), poorer OS (HR = 1.68, 95% CI: 1.43~1.97, *P* = 0.000), and DFS (HR = 1.74, 95% CI: 1.44~2.08, *P* = 0.000). Previous meta-analysis [29] also reported that cancer patients with high PVT1 expression had a poorer overall survival (HR = 2.07, 95% CI: 1.40-2.74, *P* = 0.000) and a worse disease-free survival (HR = 2.10, 95% CI: 0.96-3.23, *P* = 0.000). Therefore, by detecting the expression of lncRNA PVT1 in tumor tissue, we can have a comprehensive analysis and evaluation of the patient's condition and prognosis and provide a reference for the realization of individualized treatment.

Some limitations in our meta-analysis also should be taken into account. Firstly, the cut-off value for distinguishing low and high lncRNA PVT1 expression reported in included studies was different. Secondly, we searched databases in English and Chinese so that some relevant publications could have been ignored. Thirdly, studies included in our meta-analysis are from China; more other multicenter clinical studies from other countries should be conducted to confirm these results.

In conclusion, higher expression level of lncRNA PVT1 is significantly associated with GC patients' gender, invasion depth, poorer OS, and worse DFS. lncRNA PVT1 might act as a novel predictive biomarker of poor prognosis and clinicopathological characteristics for gastric cancer.

## Figures and Tables

**Table 1:** The characteristics of included studies.

Study	Country	Tumor stage	Sample size	Cut-off value	Follow-up (months, mean)	Detection methods	NOS score
Ding 2014	China	I-IV	31	T/N > 1	NR	qRT-PCR	7
Kong 2015	China	I-IV	80	Mean	NR	qRT-PCR	8
Yuan 2016	China	I-IV	111	Mean	NR	qRT-PCR	7
Xu 2017	China	I-IV	190	Mean	32.34	qRT-PCR	8
Huang 2017	China	I-IV	68	Mean	NR	qRT-PCR	8
Chen 2017	China	I-IV	187	NR	26.44	qRT-PCR	7
Niu 2020	China	I-IV	50	NR	NR	qRT-PCR	7
Wang 2020	China	I-IV	30	NR	NR	qRT-PCR	7

NR: not reported; qRT-PCR: quantitative real-time PCR.

## Data Availability

All data and analytic methods are available by e-mail request to the corresponding authors when required.
